# Supplementary material for: Engaging inexpensive hands-on activities using Chlamydomonas reinhardtii (a green micro-alga) beads to teach the interplay of photosynthesis and cellular respiration to K4–K16 Biology students
Source: PeerJ. 2020 Aug 25;8:e9817. doi: 10.7717/peerj.9817 (PMC7453928; doi:10.7717/peerj.9817)
Supplement: Supplemental Information 1 [file peerj-08-9817-s001.docx]

**Glimpses of dissemination of our educational outreach Plant-BLOOME project**

**Plant Biology 2019 links**

<https://www.facebook.com/uwgmitralab/photos/a.1624648821137916/2385634245039366/?type=3&theater>

**Plant Biology 2019 newsletter**

<https://www.facebook.com/uwgmitralab/photos/a.1624648821137916/2385634138372710/?type=3&theater>

**School Visits**:

<https://www.facebook.com/pg/uwgmitralab/photos/?tab=album&album_id=2261568770779248>

Governor's Honors Program, June 28th 2019, Berry College, Rome, GA

<https://www.facebook.com/pg/uwgmitralab/photos/?tab=album&album_id=2334916276777830>

Talk at the Innovations in pedagogy May 14th 2019

https://www.facebook.com/pg/uwgmitralab/photos/?tab=album&album_id=2337367579866033

Bremen Middle School Trips, May 6th & May 9th 2019

<https://www.facebook.com/pg/uwgmitralab/photos/?tab=album&album_id=2286077728328352>

Bremen High School visits on April 19th & April 26th 2019

<https://www.facebook.com/pg/uwgmitralab/photos/?tab=album&album_id=2276878015914990>

East Coweta Middle School visits in April 15th and April 22nd, 2019

<https://www.facebook.com/pg/uwgmitralab/photos/?tab=album&album_id=2274292776173514>

UWG Biology representation at Scholars Day April 2nd, 2019

<https://www.facebook.com/pg/uwgmitralab/photos/?tab=album&album_id=2261568770779248>

Victoria's lab experiment during the spring break, April 1st 2019

<https://www.facebook.com/pg/uwgmitralab/photos/?tab=album&album_id=2260862400849885>

Lithia Spring High School Visits from March 27th-March 28th 2019

<https://www.facebook.com/pg/uwgmitralab/photos/?tab=album&album_id=2258637567739035>

The Heritage School visits, March 22nd and April 12th 2019

<https://www.facebook.com/pg/uwgmitralab/photos/?tab=album&album_id=2268394893429969>

Camp Creek Middle school Visit March 20th- March 25th 2019

<https://www.facebook.com/pg/uwgmitralab/photos/?tab=album&album_id=2257468817855910>

Southern sectional American Society of Plant Biologists meeting March 16th-March 18th 2019

<https://www.facebook.com/pg/uwgmitralab/photos/?tab=album&album_id=2252708734998585>

Trip to Carrollton Junior High school, March 14th-15th 2019

<https://www.facebook.com/pg/uwgmitralab/photos/?tab=album&album_id=2251027385166720>

Crabapple Middle School Visit, 12th March 2019

<https://www.facebook.com/pg/uwgmitralab/photos/?tab=album&album_id=2337364076533050>

Carrollton High School Visit, March 5th-March 6th 2019

<https://www.facebook.com/pg/uwgmitralab/photos/?tab=album&album_id=2337361486533309>

**NSTA Newsletter links:**

<https://www.facebook.com/uwgmitralab/photos/a.1624648821137916/2119499251652868/?type=3&theater> (first page)

<https://www.facebook.com/uwgmitralab/photos/a.1624648821137916/2119499338319526/?type=3&theater> (second page)
