# Supplementary material for: Engaging inexpensive hands-on activities using Chlamydomonas reinhardtii (a green micro-alga) beads to teach the interplay of photosynthesis and cellular respiration to K4–K16 Biology students
Source: PeerJ. 2020 Aug 25;8:e9817. doi: 10.7717/peerj.9817 (PMC7453928; doi:10.7717/peerj.9817)
Supplement: Supplemental Information 2 [file peerj-08-9817-s002.docx]

**Generic names of items required for the photosynthesis/cellular respiration lab**

**Important notes to read before you proceed:** You need to harvest 50 mL of dense *Chlamydomonas* culture having a cell density of 20-21 x10^6^ cells/mL to make approximately 120-180 beads. Please calculate the cell density of the culture to determine the volume of culture you need to spin down in a benchtop centrifuge to harvest 250-395 x 10^6^ cells. To a cell pellet volume of 1.25- 1.5 mL, add 4-5 mL of 2% sodium alginate to get a final volume of 6-7 mL algae cell pellet‐sodium alginate suspension. To prepare 10 algae bead bracelets, each 5 inches long and containing 10-12 beads, you would need to make approximately 100-120 beads. Always make extra beads and store them for future use for 2-5 days in the fridge. Add 8-9 drops of the bicarbonate indicator solution to 3.5 ml- 4 mL of tap water inside the bracelets. For algae bead necklaces each of 6-7 inches long, you will need 20-35 beads per necklace. Add 10-11 drops of the pH indicator bicarbonate indicator to 4.5 ml- 5.5 mL of tap water inside the algal bead necklaces. For conducting the experiment in small 5.5 mL glass vials, use 2.5 mL of tap water, 8-10 beads and 125 microliters of the 0.02% phenol red solution.

1. 200-300 mL of dense healthy culture of *Chlamydomonas reinhardtii* wild type strain (4A+ [CC-4051 4A+ mt+] or CC4533 [CC-4533cw15mt-])

2. TAP or HSA growth media for growing Chlamydomonas .

3. YA slant for maintaining *Chlamydomonas* strain stocks on agar media. Alternatively, you can make your own TAP-agar media plates to maintain *Chlamydomonas* strains.

4. 100 mL of 2% sodium alginate solution [Note: sodium alginate is a viscous liquid when dissolved at 2%]

5. Pre-chilled 600 mL of 3% CaCl_2_

6. Graduated measuring cylinders

7. P1000 and P200 micropipettes

8. Micropipette tips (that can be fitted to P1000 and P200 micropipettes)

9. 50 mL Falcon tubes

10. Tea strainer or oil strainer or any strainer with fine mesh or coffee filters

11. Plastic spoons

12. Plastic transfer pipettes

13. Tap water

14. 500 mL or 1000 mL beakers

15. Screw capped 500 ml or 1000 mL bottles for chemical solution storage

16. pH test strips

17. Bicarbonate indicator solution

18. Petri dishes

19. Funnels

20. Magnetic stirrer and stir bars

21. Aluminum foil to cover the mouth of the algae culture flasks

22. Flexible tygon tubing (dimensions: inner diameter: 3/8 in.; outer diameter: 5/8 in.)

23. Colorful yarn from arts/craft stores like Hobby Lobby

24. 1L and 50 mL glass Erlenmeyer flask to culture algae

25. Inoculating loops for inoculating algae media

26. Ruler

27. Scissor

28. 1.5 mL Eppendorf tubes

29. 5.5 ml screw capped glass vials (optional; if you are not planning to use algae bead bracelets)

30. Hemocytometer (optional; match the green color of beads with that in the figures in the manuscript)

31. Phenol red indicator solution

Additionally, the lab requires few simple equipment like bench top centrifuge, an orbital open- air shaker for shaking and aerating algae culture flask, a weighing balance for weighing chemicals, a refrigerator for storing CaCl_2_ solution and a Bunsen burner. pH meter would be required to accurately measure pH in glass vials but is not required for algae bead bracelets as pH strips are used to measure pH of the water inside the bracelets. Cool white fluorescent lights are required to grow algae. To simulate darkness, bracelets or vials can be kept inside a cabinet drawer; alternatively, one can keep them covered with alumina foil on the bench) The color change of the algae bead bracelets and glass vials can be photographed with a cell phone camera.

**Information for ordering specific materials required for the photosynthesis/cellular respiration lab.**

**Disclaimer:** You can find the generic names of the lab items from pages 1-2. Please feel free to use alternative vendors for the lab items or you can use similar items that you have in your lab/classroom. The vendors and specific catalog numbers of items given below are just suggestions.

| **Materials** | **Vendor** | **Catalog # /website** |
| --- | --- | --- |
| CC-4051 4A+ mt+ strain (wild type strain) | Chlamydomonas Resource Center | <https://www.chlamycollection.org/products/strains/> |
| CC-4533cw15mt- (alternative wild type strain) | Chlamydomonas Resource Center | <https://www.chlamycollection.org/products/strains/> |
| HSA medium | Chlamydomonas Resource Center | <https://www.chlamycollection.org/products/media/> |
| YA slant | Chlamydomonas Resource Center | <https://www.chlamycollection.org/products/media/> |
| Hutner’s trace elements | Chlamydomonas Resource Center | <https://www.chlamycollection.org/products/media/> |
| Sodium Alginate, low viscosity | Fisher Scientific | 50-145-3340 |
| Calcium Chloride dihydrate | Fisher Scientific | C79-500 |
| Basix™ Microcentrifuge Tubes with Standard Snap Caps, 1.5 mL | Fisher Scientific | 02-682-002 |
| Fisherbrand™ Tygon S3™ E-3603 Flexible Tubing | Fisher Scientific | 14-171-133; dimensions: inner diameter: 3/8 in.; outer diameter: 5/8 in. |
| Rainbow Bridge Crafter's Secret Cotton Yarn, color 136 | Hobby Lobby | <https://www.hobbylobby.com/Yarn-Needle-Art/Yarn/Rainbow-Bridge-Crafter's-Secret-Cotton-Yarn/p/138162> |
| Hot Spot Crafter's Secret Cotton Yarn, COLOR 122 | Hobby Lobby | <https://www.hobbylobby.com/Yarn-Needle-Art/Yarn/Hot-Spot-Crafter's-Secret-Cotton-Yarn/p/138156> |
| Fisherbrand™ Disposable Inoculating Loops and Needles | Fisher Scientific | 22-363-601 |
| **Materials** | **Vendor** | **Catalog # /website** |
| Bicarbonate Indicator Solution, 30 mL Vial | Carolina Biological | C150015 (Note: This item is not listed separately on the vendor’s website. It is a component of the Algae Bead Photosynthesis Kit Item # 206100. It can be purchased separately by calling customer service directly.) |
| Hausser Scientific Bright-Line™ Counting Chamber/  Hemocytometer | Fisher Scientific | 02-671-51B |
| Phenol Red Indicator Solution, 0.02% w/v | Fisher Scientific | S25464 |
| Fisherbrand™ Disposable Graduated Transfer Pipettes | Fisher Scientific | 13-711-9AM |
| Ricca Chemical pH Test Strips; pH 0-14 | Fisher Scientific | 88-801 |
| Ricca Chemical pH Test Strips; pH 2-9 | Fisher Scientific | 88-841 |
| Fisherbrand™ Class B Clear Glass Threaded Vials with Closures Packaged Separately | Fisher Scientific | 03-339-21C |
| Pyrex™ Narrow-Neck Heavy-Duty Glass Erlenmeyer Flask, 50 mL | Fisher Scientific | 10-040C |
| Pyrex™ Narrow-Neck Heavy-Duty Glass Erlenmeyer Flask, 1 L | Fisher Scientific | 10-040K |
| Falcon 50 mL Conical Centrifuge Tubes | Fisher Scientific | 14-959-49A |

**Cost comparison of our Photosynthesis Lab with that using the Bio-Rad kit.**

| **Materials** | **Vendor** | **Catalog # /website and price** |
| --- | --- | --- |
| CC-4051 4A+ mt+ strain (wild type strain) | Chlamydomonas Resource Center | <https://www.chlamycollection.org/products/strains/>  $20 |
| HSA medium | Chlamydomonas Resource Center | <https://www.chlamycollection.org/products/media/>  $30 (10 L stock of medium) (will last for several years as we need only 50 mL of dense culture to get 120-180 beads) |
| Sodium Alginate, low viscosity, 500 grams | Fisher Scientific | 50-145-3340; $134 (will last for 30- 40 years) |
| Calcium Chloride dihydrate, 500 grams | Fisher Scientific | C79-500; $151 (lasts for several years) |
| Basix™ Microcentrifuge Tubes with Standard Snap Caps, 1.5 mL, pack of 500 | Fisher Scientific | 02-682-002; $25/pack |
| Fisherbrand™ Tygon S3™ E-3603 Flexible Tubing; 50 ft long | Fisher Scientific | 14-171-133; $ 211 (you can make 120 bracelets; each 5 inch length). dimensions: inner diameter: 3/8 in.; outer diameter: 5/8 in. |
| Rainbow Bridge Crafter's Secret Cotton Yarn, color 136 | Hobby Lobby | <https://www.hobbylobby.com/Yarn-Needle-Art/Yarn/Rainbow-Bridge-Crafter's-Secret-Cotton-Yarn/p/138162>  $2.29/yarn roll. Two-three yarn rolls are enough for making 120 bracelets. |
| Fisherbrand™ Disposable Inoculating Loops and Needles (case of 250) | Fisher Scientific | 22-363-601; $81.30 (Teacher will inoculate cultures. Hence this will also last for several years) |
| Bicarbonate Indicator Solution, 30 mL per vial | Carolina Biological | C150015 (Note: This item is not listed separately on the vendor’s website. It is a component of the Algae Bead Photosynthesis Kit Item # 206100. It can be purchased separately by calling customer service directly.) price per unit is $5.05. 30 mL per vial. This will also last for at least 2-3 years. 3 bottles is more than enough for 3-4 years. |
| Phenol Red Indicator Solution, 0.02% w/v, 500 mL | Fisher Scientific | S25464; $11.25; This will last for several years as we need only few drops. |
| **Materials** | **Vendor** | **Catalog # /website and price** |
| Fisherbrand™ Disposable Graduated Transfer Pipettes, pack of 500 | Fisher Scientific | 13-711-9AM; $57/pack of 500; Lasts for 2-3 years |
| Ricca Chemical pH Test Strips; pH 0-14; 100 strips per pack | Fisher Scientific | 88-801; $41.75 / Each |
| Fisherbrand™ Class B Clear Glass Threaded Vials with Closures Packaged Separately, pack of 144 | Fisher Scientific | 03-339-21C; $80.45 / Pack of 144; you can wash these vials and reuse them. |
| Pyrex™ Narrow-Neck Heavy-Duty Glass Erlenmeyer Flask, 50 mL | Fisher Scientific | 10-040C; $114.44 / Pack of 12; reusable |
| Falcon 50 mL Conical Centrifuge Tubes | Fisher Scientific | 14-959-49A; $418.50 / Case of 500 (will last for several years) |

Many of the above stated items will last for several years and some are re-usable. Even the bracelet tubing can be flushed out, washed and reused. We can serve more than 1100 students. Additionally, many of these items can be found at a cheaper price from other vendors, if one researches the market.

Our total investment to start this lab and serve 1100 students (we have served 947 students in the BLOOME project (see Discussion section) and, additional 200 students were served in fall 2019 in one year): $1398 approximately (add up the numbers given above and you will arrive at this value).

BioRad Kit serves 24 workstations (does not specify how many students per workstation). Let’s assume there are 4 students per workstation. Hence total number of students served = 96 students. Price of the Bio-Rad Photosynthesis and Cellular Respiration Kit for General Biology kit (in vials) (catalog #: #12005534EDU) = $181.25

If I want to serve 1100 students in one year with the Bio-Rad kit, I will need approximately 12 kits: the cost is $181.25 X 12= $2175 .

Hence our protocol is inexpensive compared to that of Bio-Rad kit. Moreover, by investing $1398, we can keep serving large population of students over longer period as many items are re-usable and have long shelf lives.

**TAP medium recipe to grow *Chlamydomonas*.**

From Gorman, D.S., and R.P. Levine (1965) *Proc. Natl. Acad. Sci. USA* **54**, 1665-1669.

**Original 20X Beijerinck recipe is:**

NH_4_Cl: 8 grams/L

CaCl_2_.2H_2_O: 1 grams/L

MgSO_4_ .7H_2_O: 2 grams/L

**We are using 40X Beijerinck as a stock in our research lab with a slightly modified recipe.** See the given recipe below and follow it.

Mwt: Molecular weight

 Make the following stock solutions:

1. **TAP salts (TAP stock nutrients/FBS solution [Filner’s Beijerinck solution X40]**

| NH_4_Cl (280mM) |  | 15.0 g |
| --- | --- | --- |
| (Mwt= 53.49) |  |  |
| MgSO_4_ .7H_2_O (16.2 mM) (Mwt= 246.47) |  | 4.0 g |
| CaCl_2_ .2H_2_O (18.10 mM) (Mwt= 147.02) |  | 2.65 g |
| water to 1 liter |  |  |

**2. Phosphate solution (1M Potassium phosphate solution, pH7)**

| \| K_2_HPO_4_ (Mwt= 174.18; 1.65M) \|  \| 28.8 g \| \| --- \| --- \| --- \| \| KH_2_PO_4_ (Mwt= 136.09; 1.058M) \|  \| 14.4 g \| \| water to 100 ml \|  \|  \| |  |  |
| --- | --- | --- | --- | --- | --- | --- | --- | --- | --- | --- | --- |
|  |  |  |
|  |  |  |

**Note you don’t have to adjust pH for this solution as it automatically gives a pH of 7-7.2 when mixed in the above stated ratio.**

**3. Hutner’s trace element (X 1000) (Note: you can purchase this solution from Chlamydomonas Resource center instead of making it; see Table S1)**

**Dissolve the following salts in order in 800 mL of E-pure water—dissolve each fully before adding the next**

| **Salt** | **Molecular Weight** | **Final concentration in 1L** | **Amount to be added in 1L** |
| --- | --- | --- | --- |
| FeSO_4_.7H_2_O | 278.01 | 18 mM | 4.99 grams |
| ZnSO_4_ .7 H_2_O | 287.56 | 76.5mM | 22 grams |
| H_3_BO_3_ | 61.83 | 184 mM | 11.4 grams |
| MnCl_2_.4H_2_O | 197.91 | 25.6mM | 5.06 grams |
| CuSO_4_.5H_2_O | 249.68 | 6.3mM | 1.57 grams |
| (NH_4_)_6_Mo_7_O_24_. 4 H_2_O | 1235.86 | 0.89mM | 1.10 grams |
| CoCl_2_.6H_2_O | 237.93 | 6.8mM | 1.61 grams |

Bring the salt mixture to a slow boil. Add 50 grams **of disodium salt of EDTA to the boiling mixture, acid form** (Na_2_ EDTA.2H_2_O; molecular weight is 372.24; final concentration in the 1L solution is 134 mM). Add KOH pellets **(and not NaOH)** to the boiling mixture to adjust the pH to 6.5. Make up the volume to 1L with pure water after adjusting the pH to 6.5. The solution should be clear green at this point. Pour the green solution in a 1 L bottle. Close the cap not too tightly. Shake it occasionally every week. The color will slowly change to dark magenta/purplish color over time. If you see any brown precipitate, filter the solution through two layers of Whatman#1 filter paper, repeating the filtration if necessary until the solution is clear.

To make the final TAP medium, mix the following:

2.42 g Tris base/Trizma
25 ml solution #1 (salts)
0.375 ml solution #2 (phosphate)
1.0 ml solution #3 (trace elements)
1.0 ml glacial acetic acid
water to 1 liter; **pH of TAP medium should be approximately between 7- 7.2 once you make it but if it is not, adjust pH with acid/base.**

For preparing solid TAP-agar media plates to maintain *Chlamydomonas* in lab, add 15 grams of agar per liter of media after checking the pH, shake it and then autoclave. **Note:** Make sure to have a magnetic stir bar inside the agar media bottle before you autoclave the bottle. Stirring using a magnetic stirrer is required during the cooling of the hot media after autoclaving to mix agar uniformly in the media solution before pouring media plates.
