# Supplementary material for: Engaging inexpensive hands-on activities using Chlamydomonas reinhardtii (a green micro-alga) beads to teach the interplay of photosynthesis and cellular respiration to K4–K16 Biology students
Source: PeerJ. 2020 Aug 25;8:e9817. doi: 10.7717/peerj.9817 (PMC7453928; doi:10.7717/peerj.9817)
Supplement: Supplemental Information 3 [file peerj-08-9817-s003.docx]

**Class workflow for photosynthesis/cellular respiration lab.**

| **Activity #** | **Time Required** | **Can Activity be Split Across Different Classes?** |  | **Can this activity be grouped with other activities?** |
| --- | --- | --- | --- | --- |
| 1. Inoculating algae growth media to culture algae and preparing bracelet tubing and yarn braids | Inoculation takes 10 minutes. It takes 30 minutes to prepare bracelet tubing and yarn braids | Not applicable as cultures need to be started 1-2 weeks ahead of bead making. |  | No |
| 2. Algae bead making | 35-40 minutes | Yes; beads can be made one-1 day before activities 3, 4 and 5 are performed |  | Yes; Activity 2 can be performed alone or combined with Activities 3 and 4 |
| 3. Bracelet Making | 10-15 minutes | Yes, can be separated from activity 2 (see above) |  | Yes, Activities 3 & 4 can be combined |
| 4. Light adaptation and light to dark shift of algae bracelets | 3-4 hours for light adaptation on day 1 and 1.5 - 4 hours for dark shift on day 2 | Yes, can be separated from activity 2 and 5 (see above) |  | Yes, Activities 2, 3 & 4 can be combined or Activities 3 & 4 can be combined. |

**Sample pre-lab and post-lab questions for the photosynthesis /cellular respiration lab.**

**SUGGESTED PRE-LAB QUESTIONS**

1. What are the reactants in the chemical reaction of cellular respiration?

2. What are the products formed in the chemical reaction of cellular respiration?

3. What are the reactants in the chemical reaction of photosynthesis?

4. What are the products formed in the chemical reaction of photosynthesis?

5. Which is the **only** chemical reaction on Earth that can produce oxygen on a mass scale?

6. Name the energy source that drives photosynthesis.

8. Circle one of the two options: The pH of Sprite is: A. Low B. High

9. Where does photosynthesis and cellular respiration occur in living cells?

**SUGGESTED POST-LAB QUESTIONS**

1. What are the names of chemical used to solidify the algae into beads?

2. Why did we not fill the entire glass vial with TAP water in the experiment?

3. What is/are the name/s of the pH indicator/s used in the vial experiment?

4. What was the color of the tap water (with the pH indicator phenol red added) in the vials at zero time point in your experiment?

5. Phenol red turns___________________ in the presence of acid.

6. Phenol red turns _____________ in the presence of base.

7. Bicarbonate indicator turns _____________ in the presence of a base.

8. Do all the beads that you made today using the wild type *Chlamydomonas* culture have the same color?

9. Which algal beads have more cells? Why?

10. What is the color of the tap water in the light exposed-vial with algae beads after 2-3hours?

11. What is the color of the tap water in the vial with algae beads that was kept in the dark after 2-3 hours? Why?

12. You might see some algae bead float up in the vial in the light (but not in the dark set), after 2- 24 hours. Why?

13. Is there any difference in the rate of photosynthesis/respiration between the two *Chlamydomonas* strains (the wild type strain and the high –light sensitive mutant strain) after 3 hours? (**Note: Observe after every 30 minutes within your class period and take pictures**).

14. Observe the light-exposed algae bead bracelet. Which beads are photosynthesizing rapidly in the bracelet? How can you infer that?

15. Chlamydomonas is unicellular eukaryotic micro-alga. These attributes make Chlamydomonas a _______________ organism.

**A sample grading rubric**

Given below is a suggested grading rubric for pre-lab and lab assignments. Criteria for success with a scoring rubric was set as follows: All students will score an average of 8 out of a total score of 10 [Note: this can be scaled up]. Of the ten grading criteria, none of the students will score less than 7.5. If the score in any category is less than 7.5, that category will need improvement.

| **Grading Criteria** | **Students** | | | | | | | | | | |
| --- | --- | --- | --- | --- | --- | --- | --- | --- | --- | --- | --- |
|  | #1 | #2 | #3 | #4 | #5 | #6 | #7 | #8 | #9 | #10 | Average |
| 1. States Objectives clearly |  |  |  |  |  |  |  |  |  |  |  |
| 2. Understands topic background information |  |  |  |  |  |  |  |  |  |  |  |
| 3. Makes observations and asks appropriate questions |  |  |  |  |  |  |  |  |  |  |  |
| 4. Generates appropriate hypothesis |  |  |  |  |  |  |  |  |  |  |  |
| 5. Follows experimental procedures properly |  |  |  |  |  |  |  |  |  |  |  |
| 6. Collects and organizes data |  |  |  |  |  |  |  |  |  |  |  |
| 7. Applies core NGSS concepts to interpret results |  |  |  |  |  |  |  |  |  |  |  |
| 8. Asks new questions based on the obtained results |  |  |  |  |  |  |  |  |  |  |  |
| 9. Generates new hypothesis |  |  |  |  |  |  |  |  |  |  |  |
| 10. Proposes experimental plans to test the generated new hypothesis |  |  |  |  |  |  |  |  |  |  |  |
