# Supplementary figures and images for: Engaging inexpensive hands-on activities using Chlamydomonas reinhardtii (a green micro-alga) beads to teach the interplay of photosynthesis and cellular respiration to K4–K16 Biology students
Source: PeerJ. 2020 Aug 25;8:e9817. doi: 10.7717/peerj.9817 (PMC7453928; doi:10.7717/peerj.9817)

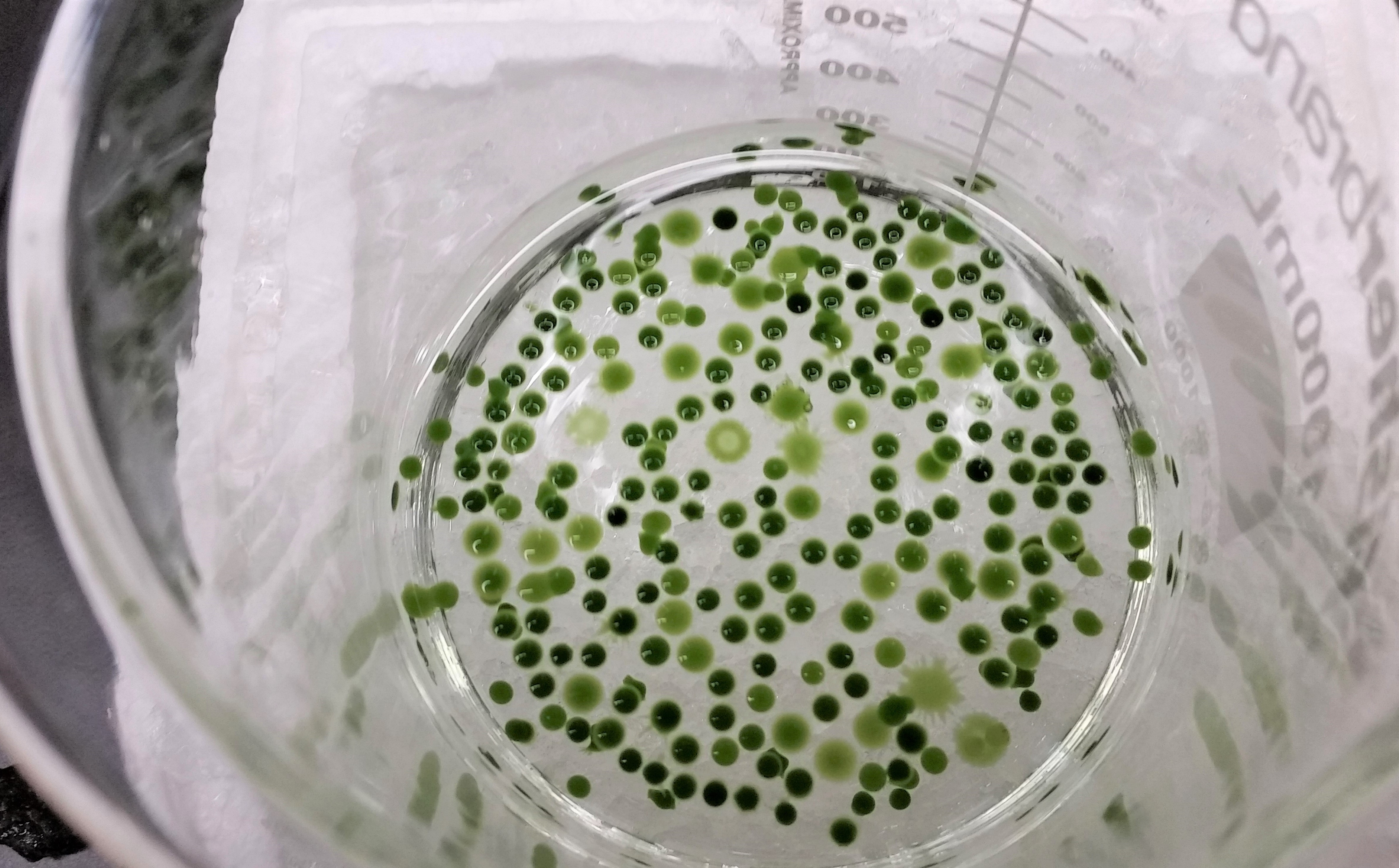

Supplement: Figure S1 — When algal cell-sodium alginate suspension is not well mixed in between pipetting and, pipetting is not performed in a steady uniform fashion, beads will assume different shapes and color because of different numbers of cells in the cell suspension drop. [file peerj-08-9817-s004.jpg]

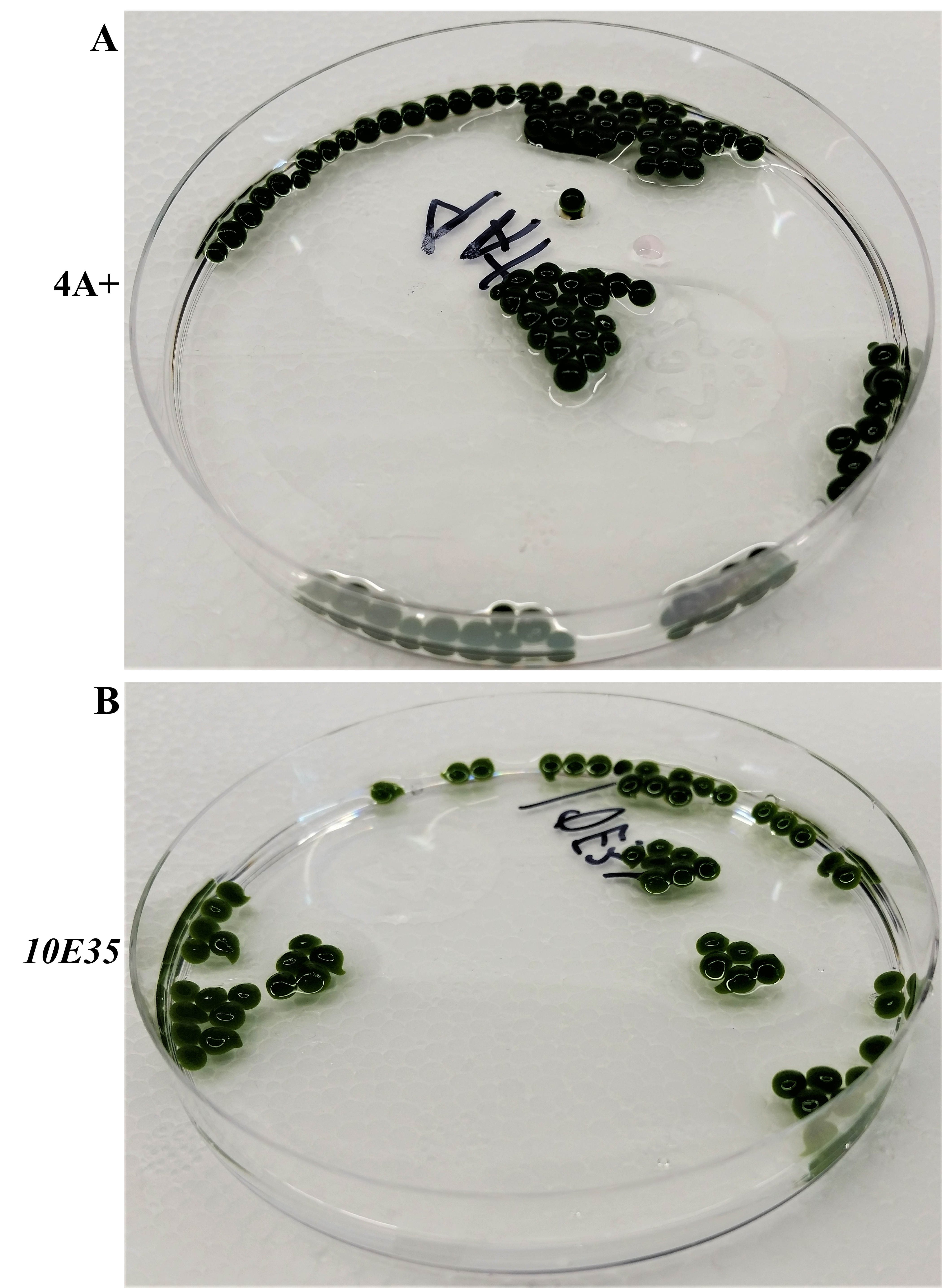

Supplement: Figure S2 — (A) 4A+ beads. (B) 10E35 beads. The beads of each strain have approximately 2 × 10^6^ cells/ bead. Note: 10E35 has less chlorophyll per cell compared to 4A+ (Nguyen et al., 2017, Article S2). Hence 10E35 beads appear slightly less dark green than the 4A+ beads. [file peerj-08-9817-s005.png]
