# Supplementary material for: Engaging inexpensive hands-on activities using Chlamydomonas reinhardtii (a green micro-alga) beads to teach the interplay of photosynthesis and cellular respiration to K4–K16 Biology students
Source: PeerJ. 2020 Aug 25;8:e9817. doi: 10.7717/peerj.9817 (PMC7453928; doi:10.7717/peerj.9817)
Supplement: Supplemental Information 8 [file peerj-08-9817-s008.pdf]

THE NGSS LIFE SCIENCE DISCIPLINARY CORE IDEAS ALIGN WITH THE 12 PRINCIPLES OF PLANT BIOLOGY FROM ASPB

LS1 From Molecules to Organisms: Structures & Processes	ASPB Principles
LS1A Structure and Function	#1, 3, 4, 5, 6, 7, 10
LS1B Growth and Development of Organisms	#1, 2, 4, 5, 6, 10, 11
LS1C Organization for Matter & Energy Flow in Organisms	#2, 3, 5, 10
LS1D Information Processing [Signaling]	#9, 11
**LS2 Ecosystems: Interactions, Energy, and Dynamics**	**ASPB Principles**
LS2A Interdependent Relationships in Ecosystems	#1, 8, 11, 12
LS2B Cycles of Matter and Energy Transfer in Ecosystem	#1, 2, 3
LS2C Ecosystem Dynamics, Functioning, and Resilience	#1, 9, 12
LS2D Social Interactions and Group Behavior	#6, 12
**LS3 Heredity: Inheritance and Variation of Traits**	**ASPB Principles**
LS3A Inheritance of Traits	#3, 4, 7
LS3B Variation of Traits	# 6, 7
**LS4 Biological Evolution: Unity and Diversity**	**ASPB Principles**
LS4A Evidence of Common Ancestry	#3, 4
LS4B Natural Selection	#3, 4, 7, 9, 11, 12
LS4C Adaptation	#3, 4, 7, 9, 11, 12
LS4D Biodiversity and Humans	#1, 6, 12

There is strong alignment with plant biology in the Physical Science and Earth & Space Science DCIs, too.

Teach with plants.

Integrate disciplinary core ideas.

Explore cross-cutting concepts.

Apply scientific practices.
