# Supplementary material for: Engaging inexpensive hands-on activities using Chlamydomonas reinhardtii (a green micro-alga) beads to teach the interplay of photosynthesis and cellular respiration to K4–K16 Biology students
Source: PeerJ. 2020 Aug 25;8:e9817. doi: 10.7717/peerj.9817 (PMC7453928; doi:10.7717/peerj.9817)
Supplement: Supplemental Information 9 [file peerj-08-9817-s009.pdf]

Proceedings of The National Conference
On Undergraduate Research (NCUR) 2017
University of Memphis, TN
Memphis Tennessee
April 6-8, 2017

Identification of a Novel Gene That Plays a Role in High Light Tolerance in the Green Micro-alga *Chlamydomonas reinhardtii*

Kevin Nguyen, Ja'von Swint, Joel Page III, Kenneth Kim, Katherine Smith,
Tai Truong, and Kasey Swilley
Department of Biology
University of West Georgia
1601 Maple Street
Carrollton, Georgia 30118 USA

Faculty Advisor: Dr. Mautusi Mitra

Abstract

Photo-autotrophic growth under different light intensities is regulated by a complex interplay of several physiological processes. *Chlamydomonas reinhardtii* is a model green micro-alga. A haploid genome, short replication time, photo-autotrophic and heterotrophic growth ability, amenability to nuclear and chloroplast transformation, and a fully sequenced genome, make it a model system for studying oxygenic photosynthesis. Our lab generated a mutant library of *Chlamydomonas* by random insertional mutagenesis using the pBC1 vector. This vector contains the *APHVIII* gene that confers resistance to paromomycin. The mutant library was screened under photo-heterotrophic and photo-autotrophic growth conditions under different light intensities leading to the isolation of 20 differentially light-sensitive mutants. One of the isolated high light-sensitive mutants is *10E35/lsr1a*. Mutant *lsr1a* is chlorophyll-deficient, hyper-sensitive to high light in photo-autotrophic and in photo-heterotrophic growth conditions and photo-bleaches on exposure to high light. *lsr1a* has a slow growth rate in photo-autotrophic condition compared to that of the wild type strain. *lsr1a* fails to grow photo-autotrophically under dim light in the presence of Rose Bengal, a singlet oxygen generator. Adapter Ligation-Mediated-PCR was performed on *lsr1a* genomic DNA. DNA sequencing of the PCR products revealed two insertion sites of pBC1 in *lsr1a*. One pBC1 insertion site is in the fourth exon of a novel functionally uncharacterized gene, to be named Cre11.g467757 *(LSR1)*. The second insertion site of pBC1 is in Cre02.g095095. Cre02.g095095 codes for a secretory cell wall protein pherophorin-C12 (PHC12) of unknown function. A strong indication that Cre11.g467757 is responsible for the *lsr1a* growth phenotype is the observation that its growth phenotype is similar to that of another uncharacterized *Chlamydomonas* Library Project (CLiP) mutant *(lsr1b),* which has a mutation in the fourth intron of Cre11.g467757. We will be presenting our physiological and molecular research on *lsr1* mutants.

Keywords: Photo-protection, *Chlamydomonas reinhardtii*, Non-photochemical quenching

1. Introduction

Chlamydomanos reinhardtii is a micro alga that is capable of photosynthesis; however, it can also grow in the dark as a heterotroph by consuming acetate as a carbon source via the glyoxylate cycle[3]. Its genome has been completely sequenced in 2007, and the alga has a short replication time between 8 and 10 hours[16, 24].

The goal of this research project is to identify molecular components involved in photo-protection. Forward genetics was the research approach used. A random nuclear DNA insertional mutant library was generated utilizing the pBC1 plasmid. Mutants were screened both in photo-heterotrophic and in photo-autotrophic growth conditions under different light conditions. Mutants which are light-sensitive to different degrees in photo-autotrophic/photo-heterotrophic growth conditions and/or were pigment-deficient in the screen, were isolated. *10E35/lsr1a* is one of the

high light-sensitive, chlorophyll (Chl) deficient mutants that was isolated during the screening. This work focuses on the physiological and molecular characterization of *lsr1a,* which has a defect in a novel functionally uncharacterized gene *LSR1* (light stress related 1).

2. Materials and Methods

2.1. Growth Of Various *Chlamydomonas* Strains

The wild-type strain of *Chlamydomonas reinhardtii*, 4A+ (UC, Berkeley), and chlorophyll deficient mutant, *lsr1a*, were maintained heterotrophically on Tris-Acetate Phosphate (TAP) agar media, in the dark or photo-heterotrophically in dim light [DL] (10-15 μmol photons $m^{-2}s^{-1}$). The dark or dim light-adapted *lsr1a* and 4A+ were used to inoculate liquid photo-heterotrophic TAP or High Salt (HS) photo-autotrophic media[26]. Liquid cultures were grown either under DL (15-20 μmol photons $m^{-2}s^{-1}$) or under high light [HL] (450-500 μmol photons $m^{-2}s^{-1}$) with constant shaking at 135 rpm at 25°C. Light intensities for all growth experiments were measured using a LI-250A Light Meter (LI-COR, Inc., Lincoln, NE).

2.2. Cell Density And Pigment Concentration Measurements

Cell density and chlorophyll (Chl) concentration per cell were determined using liquid TAP and HS cultures of 4A+ and *lsr1a*. Cultures were initially inoculated with 0.3 million cells per flask. Cell density estimations were calculated by counting the number of cells per mL of culture using Neubauer Ultraplane Hemocytometer. Photosynthetic pigments were extracted from intact cells using 80% acetone. The pigments were separated from cell debris via fixed-angle centrifugation at 16,000 x g for 5 minutes. The absorbance of the pigment-containing supernatant was then measured using a Beckman Coulter (Beckman Coulter, Brea, CA) DU730 Life Science UV-Vis Spectrophotometer. Measurements were then equated to Chl *a* and Chl *b* concentrations using Arnon[1] formulas as corrected by Melis et al.[15]

2.3. Purification And Linearization Of Pbc1 Vector And Transformation

An *Eschericia coli* clone, containing the pBC1 plasmid (carries an ampicillin resistance *[Amp^R]* and a paromomycin resistance [*APHVIII*] gene) was grown overnight in 1 L Luria-Bertini (LB) + ampicillin (100 μg/mL) media at 37°C and the *E. coli* cells were harvested by centrifugation for plasmid isolation. The plasmid purification was conducted using the QIAprep Spin Miniprep Columns (Qiagen, Valencia, CA) according to the protocol given in the technical manual. The purified plasmid was linearized by the restriction enzyme Kpn1 (NEB, Beverly, MA) according to the instructions in the product manual. The linearized pBC1 vector was used for transformation of the wild-type strain, 4A+ using the glass bead method[9] to 6000 generate random insertional mutants.

2.4. Screening Of Random Insertional Mutants

The screening of 6,000 mutants were performed using replica plating under four different growth conditions namely: Heterotrophic (TAP, dark), and photo-autotrophic growth conditions under three different light conditions (dim light [15-20 μmol photons $m^{-2}s^{-1}$], low light [80-90 μmol photons $m^{-2}s^{-1}$] and high light [450-500 μmol photons $m^{-2}s^{-1}$]. 2 μL of dense TAP cultures[24] of each of the 6000 isolated mutants and the wild type 4A+ (positive control) strain were spotted on the respective media plates as stated above and allowed to grow for a week using replica plating on each of the four media plates as stated above. Growth was monitored for 1 week. The screening resulted in the isolation of twenty light-sensitive mutants. One of these isolated mutant is *lsr1a*.

2.5. Genomic DNA Isolation From *Lsr1a* And 4A+ Strains

The *lsr1a* cells were lysed using SDS-Elution Buffer [2% SDS, 400mM NaCl, 40mM EDTA, 100mM Tris-HCl, pH 8]. Genomic DNA was extracted using Phenol:Chloroform:Isoamyl Alcohol (25:24:1) extractions by centrifugation at 12,000 x g for 20 minutes at room temperature. The extracted genomic DNA prep was digested with RNAase at 37°C for an hour to digest any RNA contaminant. After RNAase digestion, genomic DNA was extracted using

Chloroform:Isoamyl alcohol (24:1) in a microcentrifuge by centrifugation at 12,000 x g for 20 minutes. The aqueous supernatant was transferred to ice cold 100% ethanol and incubated at -20˚C overnight to precipitate the genomic DNA. The precipitated genomic DNA was sedimented by centrifugation at 12,000 x g for an hour. The DNA pellet was washed using 300-450 µL of ice cold 70% ethanol by centrifugation at 12,000 x g for 15 minutes. The supernatant was discarded and the DNA pellet was allowed to air dry for 20-25 minutes at room temperature. The pellet was dissolved in Tris-EDTA buffer [10mM Tris, 1mM EDTA, pH 8] and was placed in a 50°C water bath for 3 minutes. The genomic DNA tubes were then transferred to 4°C for 2-3 days. Concentration of genomic DNA was measured using a NanoDrop ND -1000 UV-Vis Spectrophotometer (ND-1000, Waltham, MA).

2.6. Adapter Ligation Mediated-PCR Method

Isolated genomic DNA of *lsr1a* and was digested with blunt end-cutter restriction enzymes that cut outside the pBC1 vector sequence and not within it. The preparation of the asymmetric adapter and the ligation of the genomic DNA fragments to the asymmetric adapter [sequences of the short and long strand of the asymmetric adapter are given in Table 1] were performed using the protocol given in Pollock et al., (2017)[23]. Adapter and *APHVIII*-specific primers were used on the adapter ligated-genomic DNA fragments to amplify *APHVIII*-specific flanking genomic DNA in *lsr1a*.

2.7. Gel Extraction Of DNA And DNA Sequencing

DNA gel electrophoresis of the Adapter Ligation-Mediated PCR products from *lsr1a* was performed on a 1% agarose gel. The agarose gel piece containing the PCR products were excised by a clean, sharp razor blade. Gel extraction was purified using QIAEX II Agarose Gel Extraction (Qiagen, Valencia, CA) according to the protocol given in the technical manual. The PCR products were cloned in the pCR4™-TOPO vector using the TOPO-TA cloning kit for sequencing (ThermoFisher Scientific, Waltham, MA). Purified plasmids containing the cloned PCR products were sent to UC Berkeley DNA Sequencing Facility for DNA sequencing.

2.8. Total RNA Purification

Total RNA was isolated from TAP and HS liquid cultures of *lsr1a* and 4A+ grown under dim light intensity [DL] (10-15 μmol photons $m^{-2}s^{-1}$) using TRIzol Reagent Kit (Invitogen, Carlsbad, CA) according to the protocol given in the technical manual.

2.9. cDNA Synthesis

cDNA synthesis was performed using the total RNA isolated from 4A+ and *lsr1a* cells grown in TAP media in DL, using the SuperScript III First-Strand Synthesis System (ThermoFisher Scientific, Waltham, MA) according to the protocol given in the technical manual. The cDNA was then amplified using the *LSR1*-specific primers.

3. Results

3.1. pBC1 Vector Used For Generation Of The HL-sesnitive mutant *lsr1a*

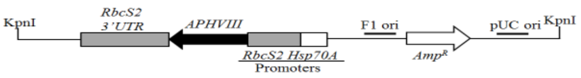

Figure 1. A schematic diagram of the linearized pBC1 plasmid used for insertional mutagenesis. The cleavage site of Kpn1 restriction enzyme, used for linearization of the vector is shown.

Figure 1. pBC1 plasmid was used as the vector for random DNA insertional mutagenesis to generate the *Chlamydomonas* mutant library. This plasmid contains both a paromomycin resistance gene *(APHVIII)* and an ampicillin resistance gene *(Amp^R)*. Paromomycin is an antibiotic which inhibits eukaryotic translation. Ampicillin is an antibiotic that prevents prokaryotic cell wall synthesis. The pBC1 vector contains dual promoters. The RbcS2 (Rubisco small subunit 2) is a constitutive promoter, and the Hsp70A (Heat shock protein 70A) is a heat/light-inducible promoter.

3.2. Comparative Growth Phenotypes of *lsr1a* and 4A+

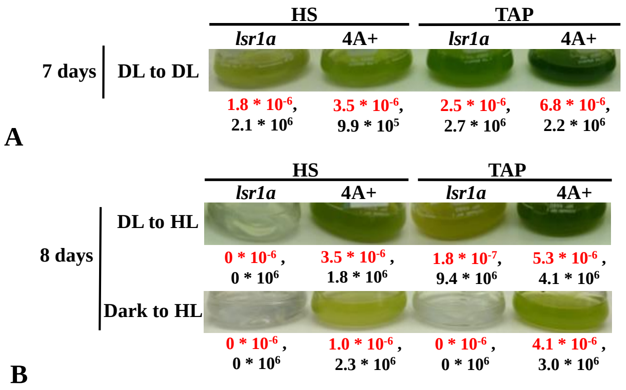

Figure 2. **(A)** Photo-autotrophic and photo-heterotrophic growth of *lsr1a* and 4 A+ (wild type) under dim light [DL] (15-20 μmol photons $m^{-2}s^{-1}$). **(B)** Photo-autotrophic and photo-heterotrophic growth of *lsr1a* and 4A+ under high light [HL] (450-500 μmol photons m^{-2} s^{-1}) when shifted from DL or from dark. The red and black numbers denote Chl concentration in nmol Chl/cell and cell density in cells/mL, respectively. Statistical error (+/- SD) was <10% of the values shown.

Figure 2. In liquid TAP media, *lsr1a* grew almost as well as the 4A+ under dim light. When *lsr1a* dim light adapted cells were transferred to high light, there was noticeable photo-bleaching in the TAP media, and *lsr1a* showed no growth in the photo-autotrophic High Salt media. When *lsr1a* dark-adapted cells were transferred to high light, it did not grow in the photo-heterotrophic or photo-autotrophic media. Under dim light conditions, the Chl per cell in *lsr1a* was approximately three-fold lower than that of 4A+ in the TAP media; the Chl per cell of *lsr1a* was about two-fold lower than that of 4A+ in the HS media. The Chl/cell in *lsr1a* is twenty-nine-fold lower than that of 4A+ in high light photo-heterotrophic growth condition. *lsr1a* fails to survive in photo-autotrophic growth conditions under high light.

3.3. Growth Phenotype of *lsr1a* and 4A+ Under Singlet Oxygen Induced-Photo-Oxidative Stress

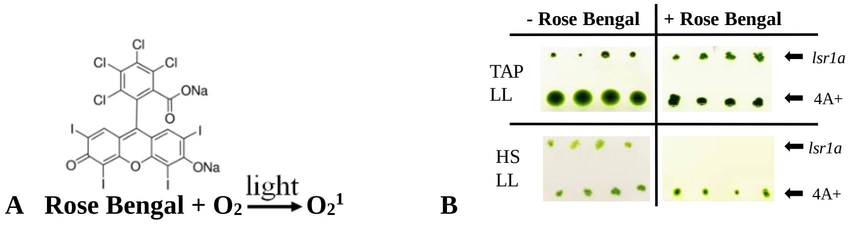

Figure 3. **(A)** An equation showing the chemical structure of Rose Bengal and its singlet oxygen (O_2^1) generating-reaction. **(B)** Photo-autotrophic and photo-heterotrophic growth of *lsr1a* and 4A+ on plates containing Rose Bengal under low light [LL] (80-90 μmol photons m^{-2} s^{-1}). A concentration 2 μM of Rose Bengal was added to the media. The media plates were incubated for four days to monitor growth.

Figure 3. **(A)** Plant and algal cells generate reactive oxygen species (ROS) in the presence of high light and oxygen. O_2^1, a type ROS, is generated in photosystems II in the chloroplast. To mimic this effect, the photo-sensitizer Rose Bengal was used to generate singlet oxygen in the presence of light and oxygen. **(B)** *lsr1a* grows much slower in

photo-heterotrophic growth conditions than the wild type on plates containing Rose Bengal. *lsr1a* fails to grow on HS plates containing Rose Bengal. This shows that the HL-sensitive photo-autotrophic growth phenotype of *lsr1a* is due to photo-oxidative stress.

Table 1. Sequences of the asymmetric adapter and the Polymerase Chain Reaction (PCR) primers used in adapter ligation-mediated PCR. The asymmetric adapter strand sequences are shown as long strand and short strand. The adapter specific primers (AP1, AP2) and the paromomycin-specific primers (PAR3, PAR6) are shown. These primers were used in the adapter ligation-mediated PCR.

Adapters/ Primers	Sequences
Long Strand (LS)	5'- GTAATACGACTCACTATAGAGGACGCGTGGTCGACGGCCCGGGCTGCT -3'
Short Strand (SS)	5'- PO3-AGC AGC CCG G – C6- NH2 -3'
AP1	5'- GTA ATA CGA CTC ACT ATA GAG G -3'
AP2	5'- ACTATAGAGGACGCGTGG -3'
PAR3	5'- GTATCGGAGGAAAAGCT -3'
PAR6	5'- GCTGTTGGACGAGTTCT -3'

3.4 Identification Of The Mutation Locus in *lsr1a*

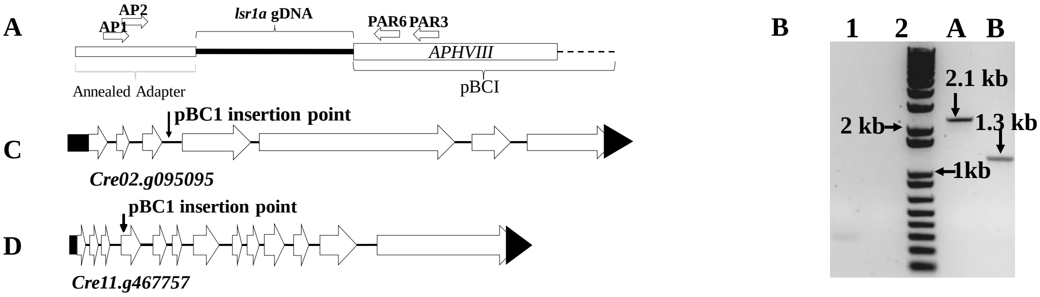

Figure 4. **(A)** A schematic diagram showing the location of the primers for Adapter Ligation-Mediated PCR to find the *APHVIII*-specific flanking genomic DNA (shown as a black line). White block arrows denote adapter ligation-mediated PCR primers **(Table 1)**. **(B)** 1% agarose gel showing adapter ligation-mediated PCR products using the AP2 and PAR6 primers **(Table 2)**. The 2.1 kb and 1.3 kb PCR products were purified from the agarose gel pieces and sent off to UC Berkeley DNA Sequencing Center. **(C)** A schematic showing the mutation locus in *lsr1a* in the fourth exon of the Cre11.g467757 gene (*LSR1*), which encodes a novel protein. **(D)** A schematic showing the mutation locus in *lsr1a* in the third intron of the Cre02.g095095, which encodes a predicted pherophorin C-12 (PHC 12). The black shapes denote UTRs, the white arrows represent exons, and the black lines denote introns. Black arrows show the insertion points.

Figure 4. The DNA sequencing of the 2.1 kb PCR product showed an insertion of the pBC1 vector in the third intron of the Cre02.g095095 gene. The DNA sequencing of the 1.3 kb PCR product showed a second insertion of the pBC1 vector in the fourth exon of the Cre11.g467757 gene. Hence *lsr1a* has two insertions of the pBC1 vector.

Table 2. Sequences of the *LSR1*-specific PCR primers used for Reverse Transcription PCR (RT-PCR). The PCR primers shown below were used to verify the presence/absence of the *LSR1* transcript in the *lsr1a*, *lsr1b* mutants and the respective wild type strains, 4A+ and cc4533.

Adapters/ Primers	Sequences
GF5	5'- GCC TGC TGC TTT TAG TGA CGC T -3'
GR8	5'- GGA CAA CCC CTG CTG CTA CAT GA -3'
G7F	5'- TGG TTT TCG CGC ATA AAC GTT GC -3'
G8F	5'- GTT GTC CTG CAT CTC GCA AAC AAT G -3'
G8R	5'- CAT TGT TTG CGA GAT GCA GGA CAA C -3'
G9F	5'- GAG CAG CAC ACC GCC TGT -3'
G9R	5'- ACA GGC GGT GTG CTG CTC -3'
G3F	5'- TGG CCC CGC TGG TCC TG -3'
G3R	5'- CAG AAC CAG CGG GGC CA -3'
G21R	5'- CGC TCG CTT CAG CAA GTT CAC GC -3'
G31F	5'- CAA GCG GAA AAC TGC GGC TCA G -3'

3.5. Comparative *LSR1* Transcript Analyses in *lsr1a* and 4A+ Strains

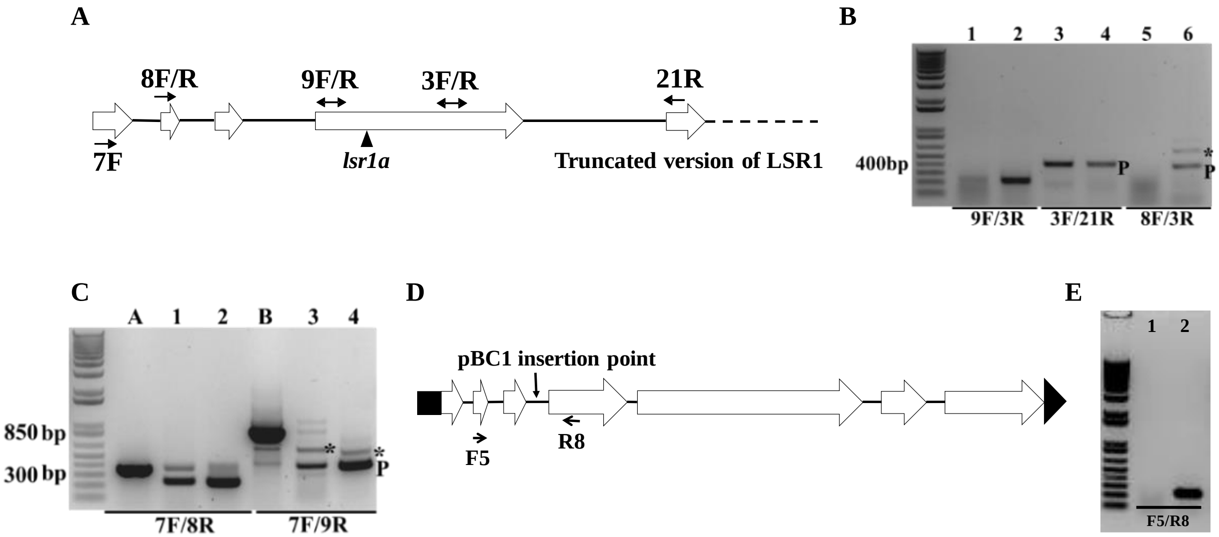

Figure 5. **(A)** A schematic of the truncated *LSR1* gene. The white arrows represent exons, and the black lines denote introns. Black dotted lines denote portion of the *LSR1* gene not represented in schematic. The black arrows denote the *LSR1*-specific PCR primers. The black triangle denotes the mutation locus in *lsr1a* in the fourth exon of the *LSR1* gene. **(B)** and **(C)** 1% agarose gels showing RT-PCR amplified products amplified from *lsr1a* and 4A+. Odd numbered lanes show transcripts generated by *lsr1a*. Even numbered lanes show transcripts generated by the wild type 4A+. Lanes A and B show PCR products amplified using the wild type genomic DNA. cDNA was prepared from DL, TAP grown cells. **(D)** A schematic of the pBC1 insertion point in the third intron of Cre02.g095095. **(E)** 1% agarose gels showing RT-PCR amplified products amplified from *lsr1a* and 4A+. Odd numbered lanes show transcripts generated by *lsr1a*. Even numbered lanes show transcripts generated by the wild type 4A+. cDNA was prepared from DL, TAP grown cells. Three independent RT-PCR reactions were conducted per each of the three independent RNA preparations.

Figure 5. **(A)** and **(B)** When primers used in PCR, spanned the mutation locus **(Table 2)**, PCR product was not generated. When primers located downstream of the mutation locus were used **(Table 2)**, a PCR product was generated. Lane 6 contains a second PCR product (*) that was not predicted by Phytozome database. This transcript includes intron 3 which led to an in-frame premature termination codon (PTC). The PCR product with the PTC (*) is

referred in the text as an unproductive transcript, and the PCR product without the premature termination codon (P) is referred in the text as the productive transcript. **(A)** and **(C)** When primers used in PCR were located upstream of the mutation locus **(Table 2)**, PCR product was generated. The results show that the splicing pattern of *LSR1* has been altered in *lsr1a*. Both the unproductive and productive products of primer set 8F/3R and 7F/9R were sequenced, and the sequencing results showed that the third intron was included and leads to an in-frame PTC. The unproductive transcript is not gDNA because the first and the second introns were spliced out but the third intron was retained in the unproductive transcript generated by the primer sets 7F/9R and 8F/3R was spliced out. Primer sets 8F/3R and 7F/9R were sequenced and is not gDNA. Sequencing data could not be included due to page number limit constraint (max of page 10 including references). The gDNA product (771 bp), unproductive (596 bp) and productive (384 bp) transcript products of primer set 8F/3R can be differentiated by their sizes in PCR. The gDNA product (902 bp), unproductive (607 bp) and productive (395 bp) transcript products of primer set 7F/9R can be differentiated by their sizes in PCR. gDNA and cDNA product sizes for primer set 3F/21R are 833 bp and 405 bp, respectively. For the primer set 9F/3R the gDNA and the cDNA product size is the same (228 bp). Hence a RT-PCR reaction should have been included as a negative control in the experiment to distinguish between the gDNA and cDNA products.

3.6. Mutation Locus In Two CLiP [Chlamydomonas Library Project] Mutants

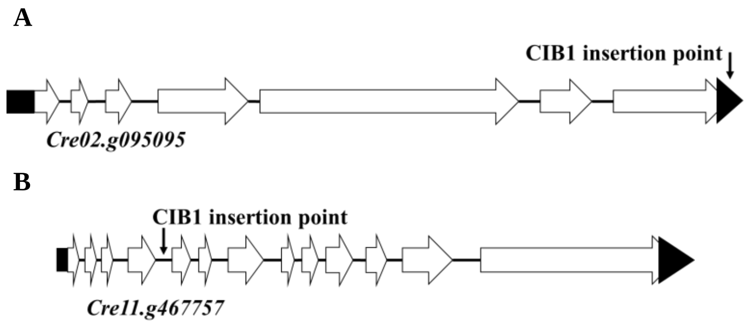

Figure 6. **(A)** A schematic diagram of the mutation locus in the CLiP mutant, LMJ.RY0402.185141 *(CLiP 185-03)*, defective in the Cre02.g095095. **(B)** A schematic diagram of the mutation locus in the LMJ.RY0402.051109 *(CLiP 05-24/lsr1b)* defective in Cre11.g467757. The black arrow shows the vector insertion point.

Figure 6. CLiP paromomycin-resistant random insertional mutants were generated using the CIB1 vector in a NSF-funded project at the Carnegie Institution of Washington (Stanford University, https://www.chlamylibrary.org/) as a global research resource for plant biologists who use *Chlamydomonas* as a model system. The CIB1 vector insertion is in the 3′ untranslated region (UTR) of Cre02.g095095. The CIB1 vector insertion in *lsr1b* is in the fourth intron of Cre11.g467757. gDNA flanking 3' and 5'- end of the CIB1 vector were amplified by PCR in both mutants and sequenced following instructions found on CLiP website (https://www.chlamylibrary.org/about). The DNA sequencing data verified the CIB1 insertion site in both CLiP mutants.

3.7. Cre02.g095095 Transcript Analysis And Growth Phenotype of the *CLiP 185-03* Under High Light

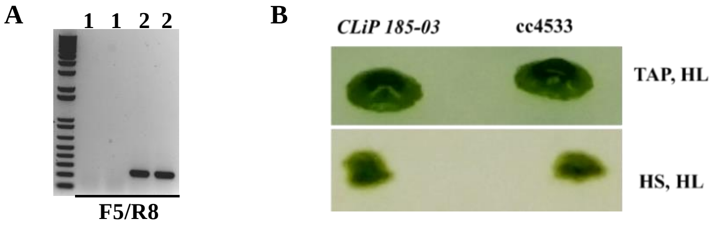

Figure 7. **(A)** 1% agarose gels showing RT-PCR amplified products amplified from *CLiP 185-03* and cc4533. Odd numbered lanes show transcripts generated by *CLiP 185-03*. Even numbered lanes show transcripts generated by the wild type cc4533. cDNA was prepared from DL, TAP grown cells. **(B)** Two independent RT-PCR reactions were conducted per each of the three independent RNA preparations. Photo-heterotrophic and photo-autotrophic growth of *CLiP 185-03* and cc4533 (wild type) under high light intensity [HL] (450-500 μmol photons m^{-2} s^{-1}).

Figure 7. **(A)** The gDNA product size is 849 bp, and the Cre02.g095095 transcript (240 bp) is absent in *CLiP 185-03* as shown in lane 1 since the primer set spans two introns. **(B)** When grown on either TAP agar media or on HS agar media, *CLiP 185-03* has a similar growth phenotype as the wild type strain cc4533. These results strongly show that *CLiP 185-03* is not high light sensitive.

3.8. Comparative Growth Studies of *lsr1a* and *lsr1b*

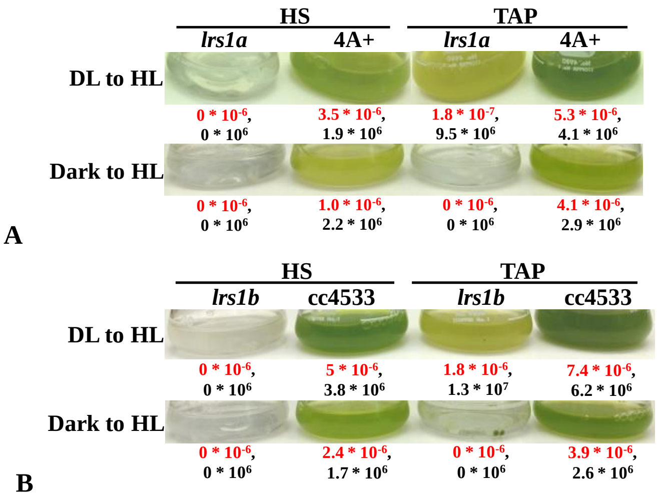

Figure 8. Photo-autotrophic and photo-heterotrophic growth of: **(A)** *lsr1a* and 4A+ and **(B)** *lsr1b* and cc4533 under high light intensity [HL] (450-500 μmol photons m^{-2} s^{-1}) when cultures were shifted from DL or dark. The red numbers denote Chl concentration in nmol Chl/cell, respectively. Statistical error (+/- SD) was <10% of the values shown.

Figure 8. Like *lsr1a*, *lsr1b* photo-bleaches in TAP media and fails to grow in the HS media during the DL to HL transition. Also like *lsr1a*, *lsr1b* fails to grow when shifted from dark to HL.

3.9. Comparative *LSR1* Transcript Analyses in *lsr1* And The Respective Wild Type Strains

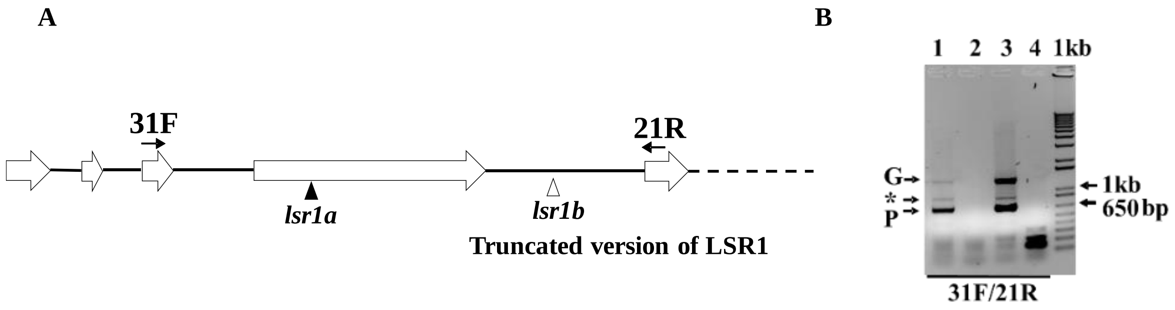

Figure 9. **(A)** A schematic diagram showing the mutation sites in the *LSR1* gene for *lsr1a* and in *lsr1b*. The white arrows represent exons, and the black lines denote introns. Black dotted lines denote other portion of *LSR1* gene not represented in schematic. The black arrows denote the *LSR1*-specific PCR primers. The black triangle denotes the mutation locus in *lsr1a* in the fourth exon while the white triangle denotes the mutation locus in *lsr1b* in the fourth

intron in the *LSR1*. **(B)** A 1% agarose gel showing the RT-PCR amplified products from *lsr1* and the respective wild type strains: Lane 1: cc4533; Lane 2: *lsr1b*; Lane 3: 4A+; Lane 4: *lsr1a*. cDNA was prepared from DL, TAP grown cells. PCR Primer sequences are given in Table 2. The sizes of the gDNA product, the unproductive and productive cDNA products are 1302 bp, 874 bp and 662 bp, respectively. Three independent RT-PCR reactions were conducted per each of the three independent RNA preparations.

Figure 9. When primers spanning the third and fifth exon were used for PCR, neither *lsr1a* nor *lsr1b* generated a product; however, the wild types generated *LSR1*-specific PCR products.

4. Discussion

The growth phenotype of *lsr1a* is very similar to that of an uncharacterized CLiP [Chlamydomonas Library Project] mutant, LMJ.RY0402.051109 (*lsr1b*; parent cc4533) **(Fig. 8)**. *lsr1b* has a single mutation in the fourth intron of the *LSR1* gene **(Fig. 6B)**. The uncharacterized CLiP mutant, LMJ.RY0402.185141, defective in the Cre02.g095095 gene is not HL sensitive **(Fig. 7)**. These results indicate very strongly that the defective *LSR1* is responsible for the HL sensitive-growth phenotype of *lsr1* mutants. 42% of the proteins encoded by the *Chlamydomonas* genome, and, about 100 hypothetical "green cut" proteins detected by the Phylogenomic analysis of the *Chlamydomonas* genome have no functional predictions[7]. Phytozome (http/phytozome.jgi.doe.gov) and NCBI protein BLAST show that the predicted *LSR1* protein does not have a functionally annotated domain but has some partial sequence matches to some hypothetical proteins in green algae like *Volvox* and *Gonium*. Complementation of the *lsr1* mutants with the *LSR1* genomic/cDNA will conclusively confirm the function of *LSR1* in photo-protection and will assign for the first time a functional role to this uncharacterized gene.

^{3}Chl* generated under excess light can transfer energy to O_2, generating $O_2{}^1$ predominantly at the PSII, that causes photo-oxidative damage in plants[12, 27]. Photosynthetic organisms employ diverse photo-acclimatory and photo-protective strategies to avoid, minimize, and repair photo-oxidative damage in excess light[4, 8, 14, 20]. Current research show that vascular plants and algae have distinct biochemical strategies for dynamic regulation of photosynthesis under fluctuating light[14, 20, 21]. Non-photochemical quenching (NPQ) is one of the photo-protective processes that quenches excess absorbed light energy harmlessly as heat when excitation energy exceeds the capacity for light utilization[17, 18]. Several types of light harvesting complex proteins, photo-protective carotenoids like lutein and zeaxanthin and antioxidants like tocopherol play an important role in NPQ[19, 21, 25]. *lsr1a* photo-bleaches under high light **(Fig. 2 & Fig. 8)**. This is also supported by the chlorophyll/cell data **(Fig. 2)**. *lsr1a* grows slowly photo-heterotrophically compared to 4A+ and fails to grow photo-autotrophically in low light in the presence of the singlet oxygen generator Rose Bengal **(Fig. 3)**. We hypothesize that the high light sensitivity of *lsr1a* is due to photo-oxidative stress. We hypothesize that in *lsr1a* NPQ has been affected. In future, measurements of NPQ by fluorometric assays using a pulse modulated fluorometer, and quantification of the photo-protective carotenoids by high performance liquid chromatography will help to determine if NPQ and photo-protective carotenoid levels are affected in the *lsr1* mutants.

Transcript analysis showed that the *LSR1* transcript is made in both the *lsr1* mutants but the splicing pattern has been altered **(Fig. 5B & C)**. In *lsr1a*, splicing between 3rd exon and the 4th exon is affected **(Fig. 5B & C; Fig. 9B)**. In *lsr1b*, the splicing of the 4th exon and 5th exon is not affected in spite of the mutation, but, the splicing between exon 3 and an exon 5, is affected like that in *lsr1a* **(Fig. 9B)**. Our RT-PCR analyses detected, a second transcript that is unproductively, alternatively spliced via the *LSR1* 3rd intron inclusion, in addition to the predicted productive transcript. This intron inclusion results in a premature termination codon (PTC), not predicted in Phytozome **(Fig. 5B & C, Fig. 9B)**. Preliminary analyses show that the unproductive transcript is always present (even in the mutants) **(Fig. 5B & C, Fig. 9B)**.

In *Arabidopsis* and *Chlamydomonas*, diurnal conditions, the redox state of plastoquinol and light regulate post-transcriptional alternative splicing of genes encoding circadian clock proteins, a SR splicing factor and a chlorophyll biosynthesis regulator, FLP[5, 6, 10, 22]. If an intron inclusion in alternative splicing results in an in-frame PTC more than 50 bp upstream of the last exon-exon junction, the PTC harboring-transcripts containing the "retained" intron, are degraded via the non-sense mediated mRNA decay (NMD) in cytoplasm by a mechanism called regulated unproductive splicing and translation (RUST). mRNAs harboring a "detained" intron, are contained inside the nucleus, not subject to NMD, and undergo stress response-mediated regulated splicing[2, 10, 11, 13]. The ratio of the unproductive to the productive isoform (splicing index) can be measured using quantitative PCR in the *lsr1* mutants and in the

respective wild-type strains grown under different light conditions in different growth media. These experiments will show if *LSR1* expression is regulated by light stress mediated-post-transcriptional regulated splicing.

5. Acknowledgements

This project was funded by various undergraduate student research programs at the University of West Georgia and NSF-Georgia-Alabama-LSAMP grant (NSF Award #1305041). The authors would like thank Dr. Krishna K Niyogi (UC Berkeley) for giving us the pBC1 vector and the 4A+ strain. CLiP strains were generated by Dr. Martin Jonikas' laboratory in the Department of Plant Biology at Carnegie Institution of Washington at Stanford University.

6. References Cited

1. Arnon D. 1949. Copper enzymes in isolated chloroplasts. Polyphenol oxidase in Beta vulgaris. *Plant Physiol*, 24, 1-15.

2. Boutz, P. L., Bhutkar A. and Sharp P. A. 2015. Detained introns are a novel, widespread class of post-transcriptionally spliced introns. *Genes Dev*, 29, 63–80.

3. Cahoon A. B., Timko M. P. 2000. Yellow-in-the-dark Mutants of *Chlamydomonas* Lack the CHLL Subunit of Light-Independent Protochlorophyllide Reductase. *Plant Cell*, 12, 557-559.

4. Erickson E, Wakao S. and Niyogi K. K. 2015. Light stress and photoprotection in *(Chlamydomonas reinhardtii). Plant J*, 82, 449-465.

5. Falciatore A., Merendino L., Barneche F, et al. 2005. The FLP proteins act as regulators of chlorophyll synthesis in response to light and plastid signals in *Chlamydomonas*. *Genes Dev*, 19, 176-187.

6. Filichkin S. A. & Mockler T. C. 2012. Unproductive alternative splicing and nonsense mRNAs: A widespread phenomenon among plant circadian clock genes. *Biology Direct*, 7, 20.

7. Grossman A. R., Karpowicz S. J., Heinnickel M., et al. 2010. Phylogenomic analysis of the *Chlamydomonas* genome unmasks proteins potentially involved in photosynthetic function and regulation. *Photosynth Res*, 106, 3-17.

8. Krause G. H. 1988. Photoinhibition of photosynthesis: an evaluation of damaging and protective mechanisms. *Physiol Plant*, 74, 566–574.

9. Kindle, K. L., R. A. Schnell, E. Fernandez, and P. A. Lefebvre. 1989. Stable Nuclear Transformation of *Chlamydomonas* Using the *Chlamydomonas* Gene for Nitrate Reductase. *Cell Biology*, 6, 2589-601.

10. Labadorf A., Link A., Rogers M. F. et al. 2010. Genome-wide analysis of alternative splicing in (*Chlamydomonas reinhardtii*). *BMC Genomics*, 11, 114.

11. Lareau L. F., Brooks A. N., Soergel DA et al. 2007. The coupling of alternative splicing and nonsense-mediated mRNA decay. *Adv Exp Med Biol*, 623, 190-211.

12. Ledford H. K., Chin BL and Niyogi, KK 2007. Acclimation to singlet oxygen stress in *(Chlamydomonas reinhardtii). Eukaryot Cell*, 6, 919–930.

13. Lewis B. P., Green R. E., Brenner S. E. 2003. Evidence for the widespread coupling of alternative splicing and nonsense-mediated mRNA decay in humans. *Proc Natl Acad Sci U S A*, 100, 189-192.

14. Li Z, Wakao S., Fischer B. B., Niyogi K. K. 2009. Sensing and Responding to Excess Light. *Annu Rev Plant Biol*, 60**,** 239-260.

15. Melis A., Spangfort M., Andersson B. 1987. Light-Absorption and Electron-Transport Balance between Photosystem-II and Photoystem-I in Spinach-Chloroplasts. *Photochem and Photobiol*, 45, 129-136.

16. Merchant S. S. et al. 2007. The *Chlamydomonas* Genome Reveals the Evolution of Key Animal and Plant Functions. *Science*, 318, 245-250.

17. Müller P., Li X-P, Niyogi K 2001. Non-Photochemical Quenching. A response to excess light energy. *Plant Physiol*, 125, 1558-1566.

18. Niyogi K. K. and Truong T. B. 2013. Evolution of flexible non-photochemical quenching mechanisms that regulate light harvesting in oxygenic photosynthesis. *Curr. Opin. Plant Biol*, 16, 307–314.

19. Niyogi K. K., Björkman O, and Grossman A. R. 1997. The roles of specific xanthophylls in photoprotection. *Proc. Natl. Acad. Sci. USA*, 94, 14162–14167.

20. Niyogi K. K. 2000. Safety valves for photosynthesis. *Current Opinion in Plant Biology*, 3, 455–460.

21. Peers G, Truong T. B, Ostendorf E, et al. 2009. An ancient light-harvesting protein is critical for the regulation of algal photosynthesis. *Nature*, 462, 518-521.

22. Petrillo E, Godoy Herz MA, Fuchs A, et al. 2014. A chloroplast retrograde signal regulates nuclear alternative splicing. *Science*, 344, 427–430.

23. Pollock SV, Mukherjee B., J Bajsa-Hirschel, et al. 2017. A robust protocol for efficient generation, and genomic characterization of insertional mutants of *(Chlamydomonas reinhardti)*. *Plant Methods*, 13, 22 doi: 10.1186/s13007-017-0170-x.

24. R.M. Dent et al. .2005. Functional Genomics of Eukaryotic Photosynthesis Using Insertional Mutagenesis of *(Chlamydomonas reinhardtii)*. *Plant Physiol*, 137, 545–556.

25. Sirikhachornkit A, Shin JW, Baroli I and Niyogi KK 2009. Replacement of alpha-tocopherol by beta-tocopherol enhances resistance to photooxidative stress in a xanthophyll-deficient strain of (Chlamydomonas *reinhardtii*). *Eukaryot Cell*, 8, 1648–1657.

26. Sueoka N. 1960. Mitotic Replication of Deoxyribonucleic Acid In *(Chlamydomonas reinhardtii)*. *Proc Natl Acad Sci USA*, 46, 83-91.

27. Triantaphylides C, Krischke M, Hoeberichts FA, et al. 2008. Singlet oxygen is the major reactive oxygen species involved in photooxidative damage to plants. *Plant Physiol*, 148, 960–968.
