# Supplementary material for: Engaging inexpensive hands-on activities using Chlamydomonas reinhardtii (a green micro-alga) beads to teach the interplay of photosynthesis and cellular respiration to K4–K16 Biology students
Source: PeerJ. 2020 Aug 25;8:e9817. doi: 10.7717/peerj.9817 (PMC7453928; doi:10.7717/peerj.9817)
Supplement: Table S3 — The table shows the mean pH with standard deviations based on data from three biological replicates. Control algal bracelet was not exposed to light or to darkness (zero time point). Bicarbonate indicator was used as the pH indicator in the algal bead bracelets. pH was measured using pH testing strips. Raw pH data of three biological replicates with statistical analyses can be found in https://doi.org/10.6084/m9.figshare.12344024.v1 and in the Data S1 file. Data S2 file contains the raw pH data with mean and standard deviation information. Each biological replicate had three internal replicates. [file peerj-08-9817-s012.docx]

| **Samples** | **Mean** |
| --- | --- |
| Control bracelet | 7.00±0.00 |
| Dark-exposed 4A+ algal bracelet | 6.17±0.24 |
| Light-exposed 4A+ algal bracelet | 8.83±0.24 |
