# Supplementary material for: Engaging inexpensive hands-on activities using Chlamydomonas reinhardtii (a green micro-alga) beads to teach the interplay of photosynthesis and cellular respiration to K4–K16 Biology students
Source: PeerJ. 2020 Aug 25;8:e9817. doi: 10.7717/peerj.9817 (PMC7453928; doi:10.7717/peerj.9817)
Supplement: Table S6 — The table shows the mean pH with standard deviations based on data from three biological replicates. Phenol red was used as the pH indicator in control and experimental vials. pH was measured using a pH meter. Raw pH data of three biological replicates with statistical analyses can be found in https://doi.org/10.6084/m9.figshare.12344024.v1 and in the Data S1 file. Data S2 file contains the raw pH data with mean and standard deviation information. Each biological replicate had three internal replicates. [file peerj-08-9817-s015.docx]

| **Samples** | **Average** |
| --- | --- |
| Control before dark shift | 7.37±0.06 |
| *10E35* before dark shift | 8.33±0.06 |
| 4A+ before dark shift | 8.87±0.06 |
| Control after 15 minutes of dark exposure | 7.33±0.00 |
| *10E35* after 15 minutes of dark exposure | 7.37±0.06 |
| 4A+ after 15 minutes of dark exposure | 8.73±0.06 |
| Control after 30 minutes of dark exposure | 7.37±0.06 |
| *10E35* after 30 minutes of dark exposure | 6.93±0.06 |
| 4A+ after 30 minutes of dark exposure | 8.53±0.06 |
| Control after 45 minutes of dark exposure | 7.33±0.06 |
| *10E35* after 45 minutes of dark exposure | 6.73±0.06 |
| 4A+ after 45 minutes of dark exposure | 8.53±0.06 |
| Control after 1 hour of dark exposure | 7.33±0.06 |
| *10E35* after 1 hour of dark exposure | 6.47±0.06 |
| 4A+ after 1 hour of dark exposure | 8.40±0.10 |
